# Pediatric *Rickettsia massiliae* Infection, Spain, 2023

**DOI:** 10.3201/eid3209.260035

**Published:** 2026-09

**Authors:** Jorge Martín-Trueba, Isabel Jado, María Elena Andrés-Galván, Rosa M. González-Martín-Niño, Inés Martín-Martín, Marcos López de-Felipe Escudero, María Teresa Llorente, José María Serrano-Romero, Antonio Sampedro, Raquel Andrade-Lobato, Coral García-Esteban, Judit Gil-Zamorano, María Isabel Hurtado-Gavilán, David González-Barrio

**Affiliations:** Instituto de Salud Carlos III, Madrid, Spain (J. Martín-Trueba, I. Jado, M.E Andrés-Galván, R.M. González-Martín-Niño, I. Martín-Martín, M. López-de-Felipe Escudero, M.T. Llorente, J. Gil-Zamorano, M.I. Hurtado-Gavilán, D. González-Barrio); Consortium for Biomedical Research in Infectious Diseases, Madrid (I. Martín-Martín, D. González-Barrio); Hospital Universitario Virgen de las Nieves, Granada, Spain (J.M. Serrano-Romero, A. Sampedro); Biosanitary Institute of Granada, Granada (A. Sampedro); Hospital Universitario de Móstoles, Madrid (R. Andrade-Lobato); Hospital Universitario de Getafe, Madrid (C. García-Esteban)

**Keywords:** Rickettsiosis, rickettsia, *Rickettsia massiliae*, tick-borne, spotted fever group, vector-borne infections, bacteria, molecular epidemiology, emerging diseases, zoonoses, Spain

## Abstract

Rickettsial disease caused by *Rickettsia massiliae* is rare in humans; only 6 cases have been reported since 2006. We report a case of *R. massiliae* infection in a pediatric patient in Spain with symptoms suggestive of rickettsiosis. Our results suggest that *R. massiliae* is an emerging tickborne rickettsial pathogen.

The increasing incidence and transmission of tickborne diseases is a major public health challenge. Although surveillance contributes to understanding the distribution and ecology of tickborne diseases, they remain underdiagnosed (*1*). *Rickettsia massiliae*, a spotted fever group *Rickettsia* species, is a human pathogen that was first isolated from *Rhipicephalus* spp. ticks in France in the 1990s (*2*). Since then*,* *R. massiliae* has been identified in questing and feeding ticks from a variety of animal hosts and in asymptomatic humans across regions of Europe, Africa, Asia, and limited areas of the Americas (*3*). *R. massiliae* was first recognized as a human pathogen in 2006 (*4*). Only 6 confirmed cases of *R. massiliae* caused rickettsiosis have been reported, highlighting its emergence as a tickborne rickettsial pathogen (*5*–*7*).

*R. massiliae* infection often causes clinical symptoms similar to other rickettsial infections, including fever, muscle pain, rash, hepatomegaly, scalp bedsores, neck lymphadenopathy, and eye issues such as retinal vasculitis, conjunctivitis, and vision loss (*8*). Although ticks of the genus *Rhipicephalus* are the primary vectors (*9*), the tick species responsible for the transmission of *R. massiliae* to humans has not been identified. We report a laboratory-confirmed case of *R. massiliae* infection in a patient from southern Spain. 

In January 2023, a pediatric patient was brought to medical care for a dark eschar on the right hemiscrotum, associated with pruritus, pain, and local inflammation. The patient reported travel 2 weeks before seeking medical attention to a periurban area of Granada Province, southern Spain, where he had contact with a domestic dog but no known arthropod exposure. He remained afebrile, except for a single temperature of 38°C on the day of admission. Laboratory test results were unremarkable, including a leukocyte count of 8.9 × 10^3^ cells/µL (68.4% neutrophils), platelet count of 266 × 10^3^/µL, and hemoglobin level of 13.9 g/dL. The C-reactive protein result was elevated (51.8 mg/L; reference range <5.0 mg/L). Scrotal ultrasonography revealed thickening of the tunica vaginalis with mild hyperemia, but the testes were unaffected. Serologic testing for *Borrelia burgdorferi*, *Coxiella burnetii*, and *Rickettsia conorii* yielded negative results. The patient was treated with intravenous amoxicillin/clavulanic acid (25 mg/kg/8 h for 3 d), leading to clinical improvement. Before he was discharged, a lesion scraping was collected for microbiological analysis. Patient follow-up was conducted at the Pediatric Infectious Diseases outpatient clinic (Granada, Spain); the patient was treated with oral amoxicillin/clavulanic acid (25 mg/kg/8 h for 1 wk). Twenty days after symptom onset and confirmed rickettsial infection, the patient was prescribed doxycycline (100 mg/12 h for 3 d), leading to complete resolution of symptoms and signs.

Biopsy material was submitted to the Special Pathogens Reference and Research Laboratory, National Center for Microbiology (Madrid, Spain) for the detection of tickborne pathogens. We extracted DNA from the biopsy sample by using the QIAamp Tissue Kit (QIAGEN, https://www.qiagen.com) according to the manufacturer’s instructions. We detected DNA from *Rickettsia* spp. by performing PCR amplification of fragments from the 23S–5S rRNA intergenic spacer and the outer membrane protein A (*ompA*) gene (*10*). We purified PCR amplicons and sequenced the amplicons bidirectionally by capillary electrophoresis by using BigDye Terminator chemistry on an ABI PRISM 3130 automated sequencer (Thermo Fisher Scientific, https://www.thermofisher.com). 

We generated a consensus sequence for the 23S–5S rRNA intergenic spacer (GenBank accession no. PV679725) by aligning the sequence fragments to reference sequences retrieved from the GenBank database. We determined sequence similarity by using BLASTn (https://blast.ncbi.nlm.nih.gov). The sequence shared 100% identity with *R. massiliae* strain MTU5 (GenBank accession no. AY125013) and with an *R. massiliae* isolate (accession no. PP263054) detected in a tick belonging to the *Rhipicephalus sanguineus* complex from Spain. The consensus sequence also shared >99% identity with *Rickettsia* spp. isolated from ticks collected in Romania (accession no. MG450328) and Tunisia (accession no. KF245444) and the *R. massiliae* B29 strain isolated from Spain (accession no. AY125014).

We detected *R. massiliae* DNA from a skin biopsy of a patient with signs and symptoms consistent with rickettsial infection. The sequence we generated shared 99%–100% identity with *R. massiliae* strains previously isolated from ticks in other regions of Spain and Mediterranean countries. Our findings provide evidence that *R. massiliae* is an emerging cause of human rickettsiosis and highlight the diagnostic relevance of eschars in rickettsial infections. Eschars can be clinical indicators for early recognition, enabling prompt antimicrobial therapy, improving patient outcomes, and enhancing epidemiologic surveillance of emerging rickettsial infections.

The limited number of reported cases of *R. massiliae* in humans, despite the high prevalence in *Rhipicephalus* ticks, might reflect underdiagnosis because of nonspecific clinical signs and symptoms and limited molecular testing. Limited human–tick contact might also contribute to the lack of cases, because some *Rhipicephalus* species have limited anthropophilic behavior and exposure is likely concentrated in periurban settings. Because of the complexity of pathogen–vector–host interactions and the potential for severe clinical outcomes, *R. massiliae* should be considered an emerging public health concern in Mediterranean regions.
